# Systemic and oral adverse effects of semaglutide reported in randomized controlled trials: a scoping review

**DOI:** 10.1186/s12903-026-10305-6

**Published:** 2026-11-14

**Authors:** Daniella Muknickas, João Victor Frazão Câmara, Anton Schestakow, Manuela da Silva Spinola, Débora Heller, Matthias Hannig

**Affiliations:** 1 Clinic of Operative Dentistry, Periodontology and Preventive Dentistry, Saarland University, Homburg, Saarbrücken, 66123 Germany; 2 Department of General Dentistry, University of Tennessee Health Science Center, 875 Union Ave, Memphis, Tennessee, TN 38103 USA; 3 Department of Periodontology, UT Health, 7703 Floyd Curl Dr, San Antonio, Texas, TX 78229 USA

**Keywords:** Semaglutide, Glucagon-like peptide-1 receptor agonist, Adverse effects

## Abstract

**Objectives:**

To map the systemic and oral adverse events (AEs) of semaglutide reported in randomized controlled trials (RCTs).

**Data:**

This scoping review was conducted following the PRISMA-ScR guidelines. Only english-language and RCTs were considered.

**Sources:**

A literature search was conducted in PubMed, Scopus, and Web of Science from 2019 until 2025.

**Study selection:**

Of 630 identified records (300 in PubMed, 312 in Scopus, and 18 in Web of Science), 12 studies were included after screening. The review identified systemic AEs of semaglutide, primarily gastrointestinal events like nausea (12 studies) and diarrhea (11 studies). Less frequent effects included pancreatitis (5 studies), hepatobiliary (3 studies) and gallbladder disease (3 studies), dental caries (1 study) and dry mouth (1 study). Subcutaneous administration predominating (7 studies), followed by oral (3) and mixed routes (2), over treatment durations of 21–68 weeks and 4–9 weeks of follow-up. Additionally, systemic events such as nasopharyngitis (5 studies), COVID-19 (5 studies), and metabolic or nutrition-related disorders were reported in all 12 studies. Importantly, none of the included studies directly investigated oral or dental outcomes.

**Conclusions:**

Semaglutide demonstrated an overall favorable safety profile in the included randomized controlled trials, with adverse events predominantly gastrointestinal. However, the RCTs did not provide direct evidence regarding oral health outcomes. Therefore, the available evidence is insufficient to draw conclusions about oral adverse events associated with semaglutide, highlighting the need for randomized clinical trials specifically designed to assess oral health outcomes in patients receiving GLP-1 receptor agonists.

**Supplementary Information:**

The online version contains supplementary material available at https://doi.org/10.1186/s12903-026-10305-6.

## Introduction

Obesity and type 2 diabetes mellitus (T2DM) are major global public health challenges whose prevalence has increased substantially over recent decades. In 2022, approximately 2.5 billion adults were living with overweight, including more than 890 million with obesity, while T2DM affected hundreds of millions of individuals worldwide. Both conditions are associated with substantial morbidity, premature mortality, and socioeconomic burden, underscoring the need for effective preventive and therapeutic strategies [1, 2].

Semaglutide, a glucagon-like peptide-1 (GLP-1) receptor agonist (GLP-1RA), has emerged as an pharmacological option for the management of both T2DM and obesity [3–6]. Through receptor activation, it enhances glucose-dependent insulin secretion, delays gastric emptying, and modulates central pathways involved in appetite regulation, resulting in improved glycemic control and clinically meaningful weight loss. Semaglutide has also been associated with favorable cardiometabolic effects, while experimental studies suggest additional actions involving inflammatory pathways, oxidative stress, mitochondrial function, and adipose tissue metabolism, although some of these mechanisms remain to be confirmed in humans [7].

Despite its established clinical benefits, semaglutide is commonly associated with gastrointestinal adverse events, particularly nausea, vomiting, diarrhea, and constipation [3, 8–10]. Less frequent adverse events include abdominal pain, pancreatitis, and hepatobiliary or gallbladder disorders. Nevertheless, its overall benefit-risk profile remains favorable because of its efficacy in improving glycemic control, body weight, and cardiometabolic outcomes [8–10]. These systemic effects may also have implications for oral health, either by modifying risk factors associated with obesity and diabetes or through treatment-related adverse effects, warranting further investigation.

The relationship between diabetes and periodontal disease further emphasizes the relevance of evaluating oral outcomes associated with semaglutide therapy. Diabetes is recognized as an important risk factor for periodontal and peri-implant diseases, with poor glycemic control being consistently associated with increased disease severity and impaired tissue healing. Conversely, periodontal inflammation may contribute to worsening metabolic control, supporting a bidirectional relationship between these chronic conditions. Recent evidence also indicates that T2DM alters the composition and ecological interactions of the oral microbiome, creating a dysbiotic environment that may increase susceptibility to more severe periodontal destruction [11, 12].

Therefore, medications capable of improving metabolic control may potentially influence oral health both directly and indirectly, making the evaluation of oral AEs and benefits clinically relevant. These manifestations may arise directly from neuroendocrine mechanisms in salivary glands and taste pathways, or indirectly from recurrent gastric acid exposure secondary to vomiting and reflux, changes in hydration status, and dietary modifications associated with weight loss.

Recently, there has been growing concern about less recognized oral AEs, including xerostomia, dysgeusia, mucosal discomfort, and burning sensations. These manifestations, which may affect quality of life and treatment adherence, have been insufficiently explored in research [13, 14]. Moreover, the underlying mechanisms, possibly involving GLP‑1 receptor interactions in taste pathways, remain poorly understood [4].

Although researchers have extensively evaluated the efficacy and safety of semaglutide, oral AEs have generally been reported as secondary outcomes or described inconsistently across studies. Consequently, available evidence remains fragmented, making it difficult to establish the frequency, clinical significance, and potential mechanisms underlying these manifestations. Given the rapid expansion of semaglutide use for both diabetes and obesity, a scoping review is particularly appropriate to comprehensively map the available evidence, identify knowledge gaps, and provide a foundation for future clinical and translational research.

A scoping review was selected because the purpose was to map the AEs reporting across studies, and identify evidence gaps rather than estimate a pooled treatment effect or formally grade certainty of evidence. Thus, the aim is to evaluate the systemic and oral AEs of semaglutide treatment reported in randomized clinical trials (RCTs).

The selection of only RCTs was made to focus the analysis on the highest level of evidence for evaluating the efficacy and clinical effects of the intervention, while minimizing the influence of selection bias, confounding factors, and other methodological limitations inherent to non-RCTs study designs.

## Methods

This scoping review was conducted in accordance with the PRISMA-ScR guideline [15]. The scoping review method was selected because the aim of this study was to map the broad body of literature on the topic, identify knowledge gaps, and characterize how research has been conducted, rather than to address a narrow clinical question regarding treatment effectiveness.

Studies were considered eligible if they investigated semaglutide treatment in human adults with obesity and/or T2DM. Interventions had to include subcutaneous or oral semaglutide application. Eligible studies were required to assess treatment-related AEs effects.

The study protocol is registered under https://doi.org/10.17605/OSF.IO/9G7HY.

Studies were excluded if they involved animal models, other systemic diseases, those who underwent gastric surgery, or those on other medication. Only english-language and RCTs articles were included, based on the analysis on the highest level of evidence for evaluating the efficacy and clinical effects of the intervention, while minimizing the influence of selection bias, confounding factors, and other methodological limitations inherent to non-RCTs study designs. This eligibility criterion was defined a priori to restrict the evidence mapping to AEs documented under RCTs conditions and to minimize the influence of confounding and selection bias associated with non-randomized study designs.

The literature search was performed in PubMed, Scopus, and Web of Science databases, according to similar studies [16–19] covering all publications from 2019 until 2025. The search strategy was developed by D.M and A.S. and refined in collaboration with co-authors, but was not externally peer reviewed. The reference lists from the three databases were imported into Rayyan.

Duplicates were identified by Rayyan and were assessed manually.

The two reviewers (D.M and J.V.F.C) independently screened the titles and abstracts of retrieved records and assessed the full texts of potentially eligible studies. When necessary, a third senior reviewer (M.H.) was available for arbitration.

Data were extracted using an Excel data-charting form containing the following fields: author, participants, intervention, administration, comparator, duration, follow-up, serious AEs and side effects.

## Results

**Fig. 1:**
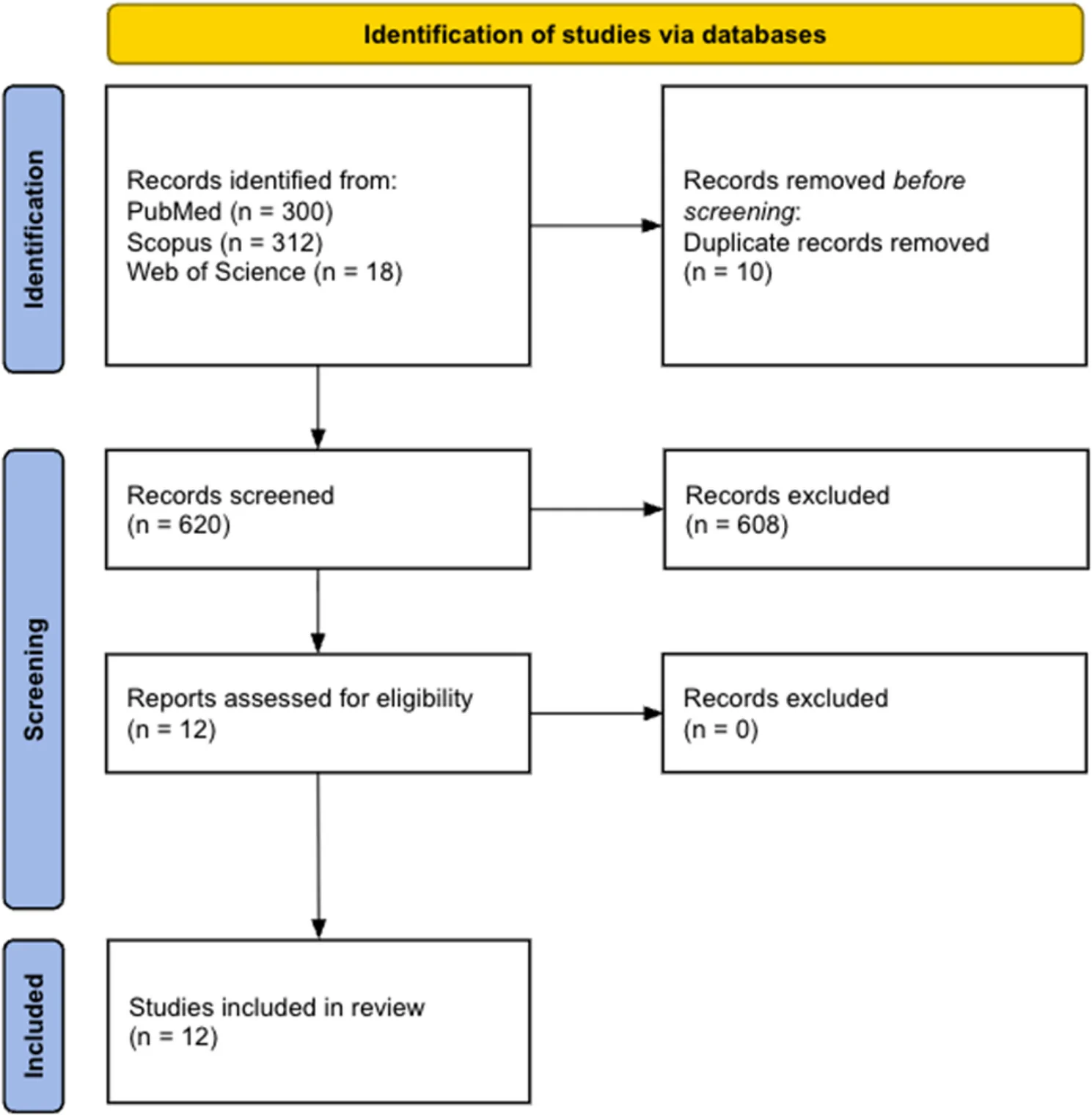
Flow chart of study selection process

A total of 630 records were identified through database searches (Fig. 1). After removing duplicates (*n* = 10), 620 were identified, and after screening titles and abstracts 608 articles were excluded, resulting in 12 studies [20–31] being included in the review.

Across the included studies, the predominant route of administration was subcutaneous injection (*n* = 7) [20, 22–24, 26, 28, 30], although oral regimens (*n* = 3) [21, 25, 29] and mixed oral–subcutaneous approaches (*n* = 2) [27, 31] were also represented. Planned treatment durations ranged from 21 to 68 weeks, with a notable clustering between 40 and 52 weeks. Follow-up periods were typically 4 to 9 weeks beyond the treatment phase.

The included studies (Supplementary material), conducted across multiple countries between 2019 and 2025, evaluated both oral and subcutaneous formulations of semaglutide in adult participants with T2DM and/or obesity. The studies varied in dosage, duration, and follow-up periods, encompassing a wide range of sample sizes and treatment protocols. Collectively, the studies assessed systemic and gastrointestinal safety outcomes, with occasional reporting of additional metabolic or hepatic effects. However, dental outcomes were reported only in 2 studies [28, 31] for semaglutide groups. One study reported an isolated case of submandibular abscess, but for the placebo group [28].

AEs were common across all studies, particularly nausea (12 studies) [20–31] and diarrhea (11 studies) [20–29, 31]. Other AEs included constipation (9 studies) [20, 22–27, 29], vomiting (8 studies) [20–24, 27, 28, 30], pancreatitis (5 studies) [20, 22–24, 28], hepatobiliary (3 studies) [20, 23, 28] and gallbladder disease (3 studies) [20, 23, 28], dental caries (1 study) [31] and dry mouth (1 study) [20]. Additionally, systemic events such as nasopharyngitis (5 studies) [20, 21, 23, 27, 31], COVID-19 (5 studies) [20, 24, 27–29], and metabolic or nutrition-related disorders were reported in all 12 studies [20–31].

Several consistent findings emerged across the dataset. Nausea [20–31] and diarrhea [20–29, 31] were the most frequently observed AEs, regardless of route of administration. High event counts were particularly evident in oral-dose studies [21, 25, 29] and in subcutaneous regimens [20, 22–24, 26, 28, 30] evaluated for weight reduction.

Events involving the hepatobiliary system were recorded [20, 23, 24, 28, 29] but remained infrequent documented hepatic events stratified by dose, and biliary disorders were reported in a small number of studies. Pancreatitis was reported [20, 22–24, 28], but absolute event counts were low. Respiratory tract infections, including nasopharyngitis and upper respiratory tract infections, and intercurrent illnesses were reported in several studies [20, 21, 23, 27, 31], likely reflecting background epidemiological patterns rather than treatment-specific effects.

Table 1 summarizes the main AEs and key safety findings reported across the included studies evaluating semaglutide use.

**Table 1:** Key safety findings and AEs reported in studies evaluating semaglutide

Author	Participants *n*	Intervention	Administration	Comparator	Duration	Follow-up	Discontinuation due to adverse events *n* (%)	Serious adverse events *n* (%)	Side effects						
									Pancreatitis *n* (%)	Gastrointestinal disorders *n* (%)				Hepatobiliary and gallbladder disorders *n* (%)	Oral/dental adverse event *n* (%)
										Constipation n (%)	Nausea n (%)	Diarrhea n (%)	Vomiting n (%)		
Aroda, 2025 [20]	Semaglutide 2 mg *n* = 60 Semaglutide 8 mg *n* = 60 Semaglutide 16 mg *n* = 62 Placebo *n* = 59	Semaglutide 2, 8, 16 mg	Subcutaneous	Placebo	Max 40 weeks	9 week -follow-up	Semaglutide 2 mg *n* = 4 (7) Semaglutide 8 mg *n* = 14 (23) Semaglutide 16 mg *n* = 11 (18) Placebo *n* = 1 (2)	Semaglutide 2 mg *n* = 4 (7) Semaglutide 8 mg *n* = 2(3) Semaglutide 16 mg *n* = 1 (2) Placebo *n* = 2 (3)	Semaglutide 2 mg *n* = 0 Semaglutide 8 mg *n* = 1 (2) Semaglutide 16 mg *n* = 0 Placebo *n* = 1 (2)	*Semaglutide 2 mg = 3% Semaglutide 8 mg = 13% Semaglutide 16 mg = 20% Placebo *n* = 5%	*Semaglutide 2 mg *n* = 20% Semaglutide 8 mg *n* = 40% Semaglutide 16 mg *n* = 40% Placebo *n* = 14%	*Semaglutide 2 mg *n* = 16% Semaglutide 8 mg *n* = 35% Semaglutide 16 mg *n* = 20% Placebo *n* = 10%	*Semaglutide 2 mg = 18% Semaglutide 8 mg = 23% Semaglutide 16 mg = 26% Placebo *n* = 7%	Hepatic events Semaglutide 2 mg *n* = 1 (2) Semaglutide 8 mg *n* = 2 (3) Semaglutide 16 mg *n* = 2 (3) Placebo *n* = 0 Gallbladder -related events Semaglutide 2 mg *n* = 2 (3) Semaglutide 8 mg *n* = 1 (2) Semaglutide 16 mg *n* = 3 (5) Placebo *n* = 0	Dry mouth *Semaglutide 2 mg = 5% Semaglutide 8 mg *n* = 3% Semaglutide 16 mg *n* = 0% Placebo *n* = 0%
Aroda, 2019 [21]	Semaglutide 3 mg *n* = 175 Semaglutide 7 mg *n* = 175 Semaglutide 14 mg *n* = 175 Placebo *n* = 178	Semaglutide 3, 7, 14 mg	Oral	Placebo	26 weeks	5 week-follow-up	Semaglutide 3 mg *n* = 4 (2.3) Semaglutide 7 mg *n* = 7 (4) Semaglutide 14 mg *n* = 13 (7.4) Placebo *n* = 4 (2.2)	Semaglutide 3 mg *n* = 5 (2.9) Semaglutide 7 mg *n* = 3 (1.7) Semaglutide 14 mg *n* = 2 (1.1) Placebo *n* = 8 (4.5)	0	NR	Semaglutide 3 mg *n* = 14 (8) Semaglutide 7 mg *n* = 9 (5.1) Semaglutide 14 mg *n* = 28 (16) Placebo *n* = 10 (5.6)	Semaglutide 3 mg *n* = 15 (8.6) Semaglutide 7 mg *n* = 9 (5.1) Semaglutide 14 mg *n* = 9 (5.1) Placebo *n* = 4 (2.2)	Semaglutide 3 mg *n* = 5 (2.9) Semaglutide 7 mg *n* = 8 (4.6) Semaglutide 14 mg *n* = 12 (6.9) Placebo *n* = 4 (2.2)	NR	NR
Capehorn, 2020 [22]	Semaglutide *n* = 290 Liraglutide *n* = 287	Semaglutide 1.0 mg	Subcutaneous	Liraglutide 1.2 mg	30 weeks	5 week- follow-up	Semaglutide 1.0 mg *n* = 33 (11.4) Liraglutide 1.2 mg *n* = 19 (6.6)	Semaglutide 1.0 mg *n* = 17 (5.9) Liraglutide 1.2 mg *n* = 22 (7.7)	Semaglutide 1.0 mg *n* = 0 Liraglutide 1.2 mg *n* = 2 (0.7)	Semaglutide 1.0 mg *n* = 17 (5.9) Liraglutide 1.2 mg *n* = 10 (3.5)	Semaglutide 1.0 mg *n* = 63 (21.8) Liraglutide 1.2 mg *n* = 45 (15.7)	Semaglutide 1.0 mg *n* = 45 (15.6) Liraglutide 1.2 mg *n* = 35 (12.2)	Semaglutide 1.0 mg *n* = 30 (10.4) Liraglutide 1.2 mg *n* = 23 (8)	NR	NR
Davies, 2021 [23]	Semaglutide 2.4 mg *n* = 404 Semaglutide 1.0 mg *n* = 403 Placebo *n* = 403	Semaglutide 1.0 mg, 2.4 mg	Subcutaneous	Placebo	68 weeks	Lifestyle intervention, followed by 7 weeks without treatment.	Semaglutide 2.4 mg *n* = 25 (6.2) Semaglutide 1.0 mg *n* = 20 (5) Placebo *n* = 14 (3.5)	Semaglutide 2.4 mg *n* = 40 (9.9) Semaglutide 1.0 mg *n* = 31 (7.7) Placebo *n* = 37 (9.2)	Semaglutide 2.4 mg *n* = 1 (0.2) Semaglutide 1.0 mg *n* = 0 Placebo *n* = 1 (0.2)	Semaglutide 2.4 mg *n* = 70 (17.4) Semaglutide 1.0 mg *n* = 51 (12.7) Placebo *n* = 22 (5.5)	Semaglutide 2.4 mg *n* = 136 (33.7) Semaglutide 1.0 mg *n* = 129 (32.1) Placebo *n* = 37 (9.2)	Semaglutide 2.4 mg *n* = 86 (21.3) Semaglutide 1.0 mg *n* = 89 (22.1) Placebo *n* = 48 (11.9)	Semaglutide 2.4 mg *n* = 88 (21.8) Semaglutide 1.0 mg *n* = 54 (13.4) Placebo *n* = 11 (2.7)	Gallbladder-related disorders Semaglutide 2.4 mg *n* = 1 (0.2) Semaglutide 1.0 mg *n* = 4 (1) Placebo *n* = 3 (0.7) Hepatic disorders Semaglutide 2.4 mg *n* = 10 (2.5) Semaglutide 1.0 mg *n* = 10 (2.5) Placebo *n* = 14 (3.5)	NR
Frias, 2021 [24]	Semaglutide 1.0 mg *n* = 469 Tirzepatide 5 mg *n* = 470 Tirzepatide 10 mg *n* = 469 Tirzepatide 15 mg *n* = 470	Semaglutide 1.0 mg	Subcutaneous	Tirzepatide 5, 10, 15 mg	40 weeks	4 week- follow-up	Semaglutide 1.0 mg *n* = 19 (4.1) Tirzepatide 5 mg *n* = 28 (6) Tirzepatide 10 mg *n* = 40 (8.5) Tirzepatide 15 mg *n* = 40 (8.5)	Semaglutide 1.0 mg *n* = 13 (2.8) Tirzepatide 5 mg *n* = 33 (7) Tirzepatide 10 mg *n* = 25 (5.3) Tirzepatide 15 mg *n* = 27 (5.7)	Semaglutide 1.0 mg *n* = 3 (0.6) Tirzepatide 5 mg *n* = 0 Tirzepatide 10 mg *n* = 2 (0.4) Tirzepatide 15 mg *n* = 2 (0.4)	Semaglutide 1.0 mg *n* = 27 (5.8) Tirzepatide 5 mg *n* = 32 (6.8) Tirzepatide 10 mg *n* = 21 (4.5) Tirzepatide 15 mg *n* = 21 (4.5)	Semaglutide 1.0 mg *n* = 84 (17.9) Tirzepatide 5 mg *n* = 82 (17.4) Tirzepatide 10 mg *n* = 90 (19.2) Tirzepatide 15 mg *n* = 104 (22.1)	Semaglutide 1.0 mg *n* = 54 (11.5) Tirzepatide 5 mg *n* = 62 (13.2) Tirzepatide 10 mg *n* = 77 (16.4) Tirzepatide 15 mg *n* = 65 (13.8)	Semaglutide 1.0 mg *n* = 39 (8.3) Tirzepatide 5 mg *n* = 27 (5.7) Tirzepatide 10 mg *n* = 40 (8.5) Tirzepatide 15 mg *n* = 46 (9.8)	Cholelithiasis Semaglutide 1.0 mg *n* = 2 (0.4) Tirzepatide 5 mg *n* = 4 (0.9) Tirzepatide 10 mg *n* = 4 (0.9) Tirzepatide 15 mg *n* = 4 (0.9)	NR
Furusawa, 2024 [25]	Semaglutide *n* = 88 Dipeptidylpeptidase-4inhibitor (DPP-4i) *n* = 86	Semaglutide 3, 7, 14 mg	Oral	Dipeptidylpeptidase-4inhibitor (DPP-4i)	24 weeks	Follow up visit at completion of 24 weeks	Semaglutide *n* = 7	NR	NR	Semaglutide 3 mg *n* = 1 Semaglutide 7 mg *n* = 2 Semaglutide 14 mg *n* = 0 DPP-4i *n* = 0	Semaglutide 3 mg *n* = 7 Semaglutide 7 mg *n* = 11 Semaglutide 14 mg *n* = 2 DPP-4i *n* = 0	Semaglutide 3 mg *n* = 1 Semaglutide 7 mg *n* = 6 Semaglutide 14 mg *n* = 0 DPP-4i *n* = 0	NR	0	NR
Kimura, 2023 [26]	Semaglutide *n* = 54 Dulaglutide *n* = 53	Semaglutide up to 1.0 mg	Subcutaneous	Dulaglutide 0.75 mg	24 weeks	Not reported	Semaglutide *n* = 4 Dulaglutide *n* = 3	**Semaglutide *n* = 1 (1.9) Dulaglutide *n* = 0	NR	Semaglutide *n* = 7 (13) Dulaglutide *n* = 1 (1.9)	Semaglutide *n* = 20 (37) Dulaglutide *n* = 4 (7.5)	Semaglutide *n* = 2 (3.7) Dulaglutide *n* = 1 (1.9)	NR	NR	NR
Lingvay, 2025 [27]	Semaglutide *n* = 340 IcoSema *n* = 341	Semaglutide 1 mg	Oral, subcutaneous	IcoSema	52 weeks	5 week- follow-up	Semaglutide 1.0 mg *n* = 9 (2.6%) IcoSema *n* = 8 (2.3%)	Semaglutide 1.0 mg *n* = 21 (6.2) IcoSema *n* = 38 (11.1)	NR	Semaglutide 1.0 mg *n* = 17 (5) IcoSema *n* = 11 (3.2)	Semaglutide 1.0 mg *n* = 39 (11.5) IcoSema *n* = 40 (11.7)	Semaglutide 1.0 mg *n* = 42 (12.4) IcoSema *n* = 38 (11.1)	Semaglutide 1.0 mg *n* = 22 (6.5) IcoSema *n* = 18 (5.3)	NR	NR
McGowan, 2024 [28]	Semaglutide 2.4 mg *n* = 138 Placebo *n* = 69	Semaglutide 2.4 mg	Subcutaneous	Placebo	52 weeks	28 week-follow-up	Semaglutide 2.4 mg *n* = 4(3) Placebo *n* = 0	Semaglutide 2.4 mg *n* = 12 (9) Placebo *n* = 6 (9)	***Semaglutide 2.4 mg *n* = 3 (2.2) Placebo *n* = 0	NR	Semaglutide 2.4 mg *n* = 1 (0.7) Placebo *n* = 0	Semaglutide 2.4 mg *n* = 1 (0.7) Placebo *n* = 0	Semaglutide 2.4 mg *n* = 1 (0.7) Placebo *n* = 0	Hepatobiliary Semaglutide 2.4 mg *n* = 1 (1) Placebo *n* = 0 Acute gallbladder Semaglutide 2.4 mg *n* = 1 (1) Placebo *n* = 0	Submandibular abscess Semaglutide 2.4 mg *n* = 0 Placebo *n* = 1 (1.4)
Wang, 2024 [29]	Semaglutide 3 mg *n* = 130 Semaglutide 7 mg *n* = 130 Semaglutide 14 mg *n* = 129 Placebo *n* = 131	Semaglutide 3, 7, 14 mg	Oral	Placebo	26 weeks	At 31 week	Semaglutide 3 mg *n* = 3 (2.3) Semaglutide 7 mg *n* = 2 (1.5) Semaglutide 14 mg *n* = 3 (2.3) Placebo *n* = 3 (2.3)	Semaglutide 3 mg *n* = 4 (3.1) Semaglutide 7 mg *n* = 2 (1.5) Semaglutide 14 mg *n* = 7 (5.4) Placebo *n* = 1 (0.8)	NR	Semaglutide 3 mg *n* = 1 (0.8) Semaglutide 7 mg *n* = 7 (5.4) Semaglutide 14 mg *n* = 3 (2.3) Placebo *n* = 0	Semaglutide 3 mg *n* = 6 (4.6) Semaglutide 7 mg *n* = 4 (3.1) Semaglutide 14 mg *n* = 8 (6.2) Placebo *n* = 2 (1.5)	Semaglutide 3 mg *n* = 4 (3.1) Semaglutide 7 mg *n* = 12 (9.2) Semaglutide 14 mg *n* = 12 (9.3) Placebo *n* = 2 (1.5)	NR	Cholelithiasis Semaglutide 3 mg *n* = 0 Semaglutide 7 mg *n* = 0 Semaglutide 14 mg *n* = 1 (0.7) Placebo *n* = 0	NR
Westergaard, 2024 [30]	31 participants were randomised and exposed to at least one of the three study products (crossover study)	Semaglutide 0.5 mg	Subcutaneous	IcoSema containing 175 U icodec (equivalent to 1050 nmol) Icodec 175 U	3 treatment periods of 5 weeks	1 follow-up visit	Semaglutide 0.5 mg *n* = 0 IcoSema *n* = 1 Icodec *n* = 0	Semaglutide 0.5 mg *n* = 0 IcoSema *n* = 0 Icodec *n* = 1 (3.6)	NR	NR	Semaglutide 0.5 mg *n* = 6 (21.4) IcoSema *n* = 14 (46.7) Icodec *n* = 0	NR	Semaglutide 0.5 mg *n* = 2 (7.1) IcoSema *n* = 11 (36.7) Icodec *n* = 0	NR	NR
Yamada, 2020 [31]	Semaglutide 3 mg *n* = 49 Semaglutide 7 mg *n* = 49 Semaglutide 14 mg *n* = 48 Placebo *n* = 49 Liraglutide 0.9 mg *n* = 48	Semaglutide 3, 7, 14 mg	Oral, subcutaneous	Placebo Liraglutide 0.9 mg	52 weeks	5 week- follow-up	Semaglutide 3 mg *n* = 1 (2) Semaglutide 7 mg *n* = 1 (2) Semaglutide 14 mg *n* = 2 (4) Placebo *n* = 0 Liraglutide 0.9 mg *n* = 0	Semaglutide 3 mg *n* = 2 (4) Semaglutide 7 mg *n* = 3 (6) Semaglutide 14 mg *n* = 0 Placebo *n* = 3 (6) Liraglutide 0.9 mg *n* = 0	0	Semaglutide 3 mg *n* = 5 (10) Semaglutide 7 mg *n* = 6 (12) Semaglutide 14 mg *n* = 6 (13) Placebo *n* = 3 (6) Liraglutide 0.9 mg *n* = 9 (19)	Semaglutide 3 mg *n* = 2 (4) Semaglutide 7 mg *n* = 5 (10) Semaglutide 14 mg *n* = 4 (8) Placebo *n* = 1 (2) Liraglutide 0.9 mg *n* = 0	Semaglutide 3 mg *n* = 4 (8) Semaglutide 7 mg *n* = 1 (2) Semaglutide 14 mg *n* = 3 (6) Placebo *n* = 1 (2) Liraglutide 0.9 mg *n* = 2 (4)	NR	NR	Caries Semaglutide 3 mg *n* = 1 (2) Semaglutide 7 mg *n* = 3 (6) Semaglutide 14 mg *n* = 1 (2) Placebo *n* = 2 (4) Liraglutide 0.9 mg *n* = 0

*NR*, not reported

* Percentages were visually estimated from Supplementary Figure 11 because numerical data were not reported in the manuscript or supplementary tables

**Reported but not related to the study

*** The value represents the sum of events reported as acute pancreatitis and pancreatitis

## Discussion

Among the RCTs included in this scoping review, semaglutide was associated with a range of reported AEs, with gastrointestinal disorders being the most frequently documented. Gastrointestinal disorders are the most frequently reported and occur with both oral and injectable formulations [20–31]. However, their frequency and severity vary according to dose, duration of treatment, and individual patient characteristics. Less frequent but clinically significant systemic AEs, such as pancreatitis [20, 22–24, 28] have been described and warrant particular attention due to their potential severity and, in the case of pancreatitis, the possibility of severe pain and life-threatening complications [20, 22–24, 28].

Hepatobiliary disorders may include hepatic and biliary conditions, which were reported in the included studies as hepatic events, gallbladder-related disorders [20, 23, 28], and cholelithiasis [20, 23, 24, 28, 29] and which can have implications for drug metabolism and overall systemic health. These systemic conditions may influence the delivery of health care, as acute abdominal pain, hepatic dysfunction, or systemic inflammation can affect clinical decision-making, appointment scheduling, medication prescription, and the patient’s tolerance to dental procedures. Respiratory infections, including COVID-19, were also reported [20, 24, 27–29], although these events are likely influenced by external factors rather than a direct pharmacological effect.

When comparing formulations, both oral and subcutaneous semaglutide demonstrate strong efficacy, but differ in tolerability and patient preference. Oral semaglutide [21, 25, 27, 29, 31] has been associated with a higher frequency of AEs, whereas injectable formulations [20, 22–24, 26–28, 30, 31] present therapeutic benefits. Longer-acting weekly injections may improve adherence due to greater convenience, while daily oral dosing requires sustained patient compliance. Recognizing the systemic nature of these AEs is essential to ensure safe, individualized care and appropriate interdisciplinary management.

Regarding oral side effects, the evidence identified in the included RCTs was limited. Isolated cases of dental caries [31] as well as dry mouth [20] were reported only in one study each, occurring across different semaglutide doses as well as in the placebo group in the case of caries. Given the low incidence, the inclusion of placebo participants, and the reliance on a single study, these findings do not allow causal inference and are most appropriately interpreted as incidental rather than treatment-related. Consequently, no consistent evidence currently links semaglutide therapy to an increased risk of dental caries. However, dental caries is a chronic process that may require prolonged periods to develop, and the relatively short follow-up periods of most included studies may have been insufficient to detect potential long-term effects.

Gastric acid is a well-established etiological factor for dental erosion [32], as its low pH promotes progressive demineralization [32–35]. Intrinsic dental erosion is defined as the non-bacterial dissolution of dental hard tissue caused by gastric acid secreted by the parietal cells of the stomach [34], which may reach the oral cavity through vomiting [20–24, 27, 28, 30].

Xerostomia, defined as the subjective sensation of dry mouth [20, 36], has been reported in association with GLP-1 receptor agonists [20]; however, this symptom does not necessarily reflect objective salivary gland dysfunction or reduced salivary flow. Although this finding were only reported in one of the studies included in this review [20], studies suggest dry mouth is associated with semaglutide use, while isolated clinical case reports have described temporally related hyposalivation [10]. Proposed mechanisms include systemic effects related to body water reduction during treatment, as well as nasopharyngitis, which is commonly reported with semaglutide and may promote mouth breathing and contribute to oral dryness [36]. Importantly, current evidence does not demonstrate a consistent or clinically relevant reduction in salivary flow attributable to semaglutide, and therefore does not support a mechanistic link between its use and dental caries. While saliva plays a protective role in acid buffering, acquired pellicle formation, and enamel remineralization [37], any discussion of caries risk in this context should remain speculative and interpreted with caution. Given that dental erosion is a cumulative process dependent on the frequency, duration, and intensity of acid exposure, short-term use of GLP-1 receptor agonists alone is unlikely to result in clinically significant dental hard tissue loss.

**Fig. 2:**
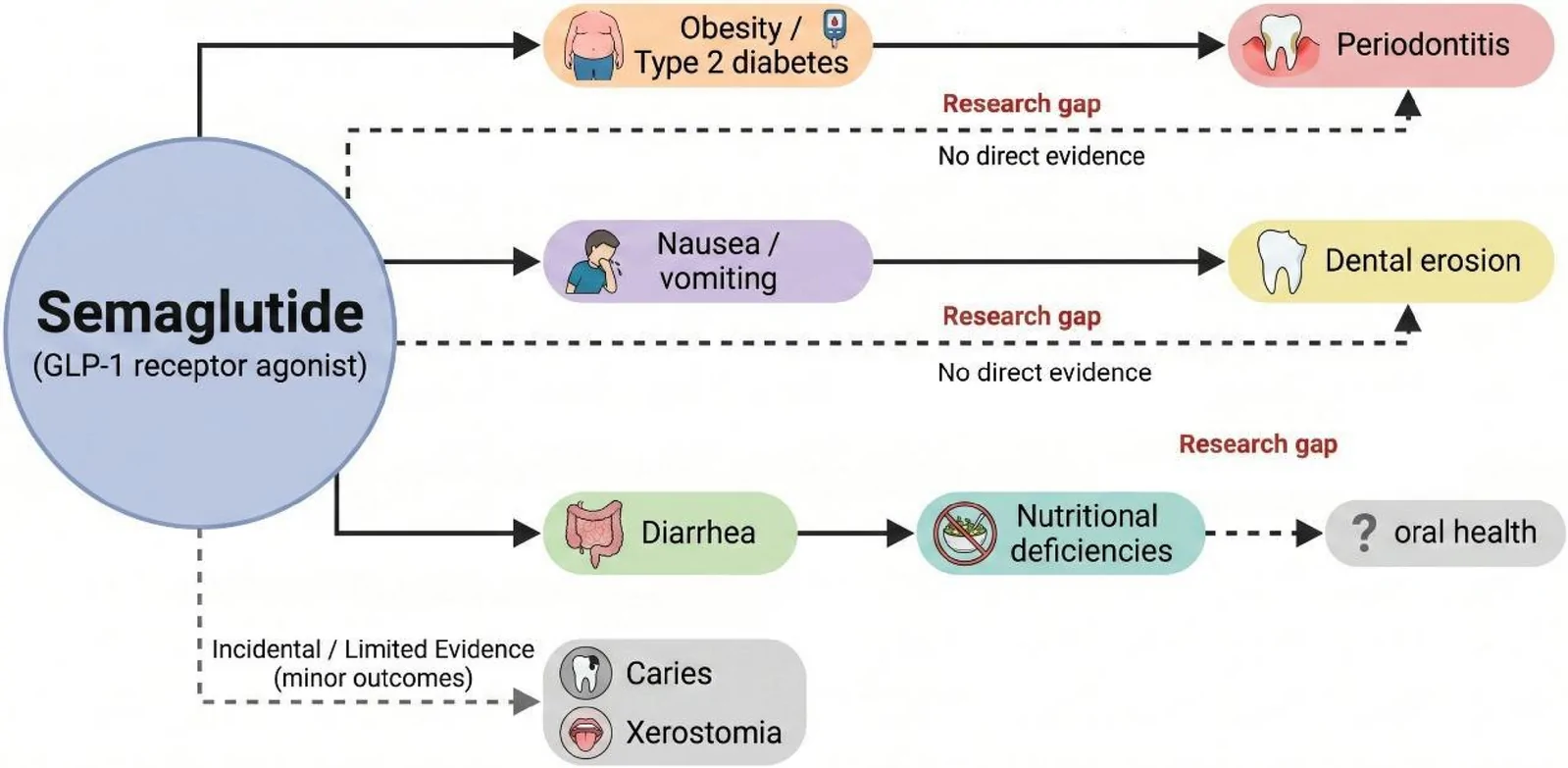
Identified research gaps (Created in BioRender. Schestakow, A. (2026) https://BioRender.com/ttj8m09)

The increasing use of injectable weight-loss medications has prompted discussion regarding potential micronutrient deficiencies. However, it is important to contextualize these considerations within the clinical profile of individuals receiving semaglutide [38], as a substantial proportion already present with periodontal alterations associated with T2DM, a well-established risk factor for periodontal disease. Conversely, by improving obesity and glycemic control, semaglutide could potentially exert beneficial effects on periodontal health by reducing established periodontal risk factors. InThe underreporting of these oral outcomes limits the ability to establish a comprehensive safety profile of semaglutide and underscores the need for studies that integrate systemic and oral health perspectives. It should be acknowledged that several of the oral signs and symptoms reported across the included studies may have been present prior to patient enrollment, and their identification during follow-up may reflect the first formal clinical documentation of pre-existing conditions rather than new-onset manifestations attributable to semaglutide use (Fig. 2).

To improve this scenario, future clinical trials and real-world studies should incorporate standardized oral health assessments as part of safety monitoring protocols. These assessments should include the evaluation of xerostomia, salivary flow rate, oral mucosal lesions, taste disturbances, periodontal status (e.g., bleeding on probing and periodontal pocket depth), dental caries, and patient-reported oral symptoms and oral health-related quality of life through validated questionnaires. Longer follow-up periods will also be essential, particularly for outcomes such as dental caries, which may require extended periods to develop or progress. In addition, prospective cohort studies incorporating standardized oral health outcomes could provide valuable evidence on the long-term oral effects of GLP-1 receptor agonists under real-world conditions.

Collaboration between endocrinologists, dentists, and clinical pharmacologists may enhance the detection and reporting of oral AEs, broadening the understanding of semaglutide’s impact beyond systemic outcomes. Furthermore, the adoption of standardized diagnostic criteria and interdisciplinary research may improve the consistency and comparability of future evidence, ultimately supporting the development of evidence-based clinical guidelines.

Other limitations should be considered when interpreting its findings. The literature search was restricted to PubMed, Scopus, and Web of Science. Although these databases provide broad coverage of the biomedical literature, the exclusion of other sources, such as Embase and LILACS, may have resulted in the omission of relevant studies, particularly those published in regional journals or indexed exclusively in these databases. A formal pilot test or calibration of the data extraction form was not performed, which may have increased the risk of inconsistent interpretation during data extraction. Additionally, oral AEs were inconsistently reported across the included studies, as they were generally assessed as secondary outcomes, and considerable heterogeneity was observed regarding study populations, treatment regimens, follow-up duration, and outcome reporting. The use of external literature to contextualize oral health outcomes also represents a limitation.Because the trials differed in population, dose, comparator, treatment duration, and adverse-event reporting, no formal comparison between administration routes was performed.

The restriction to RCTs also limits the breadth of the evidence mapped in this review, as oral AEs reported in observational studies, case reports, pharmacovigilance databases, or other study designs were not captured. Therefore, the findings should be interpreted specifically as a mapping.

Because this study was conducted as a scoping review, no formal critical appraisal of the methodological quality or risk of bias of the included studies was performed, in accordance with the exploratory purpose of this review design. Therefore, the findings should be interpreted as a comprehensive mapping of the available evidence rather than as an assessment of the certainty, strength, or quality of the evidence.

## Conclusion

Semaglutide demonstrated an overall favorable safety profile in the included randomized controlled trials, with adverse events predominantly gastrointestinal. However, the included studies did not provide direct evidence regarding oral health outcomes. Therefore, the available evidence is insufficient to draw conclusions about oral adverse events associated with semaglutide, highlighting the need for randomized clinical trials specifically designed to assess oral health outcomes in patients receiving GLP-1 receptor agonists.

## Supplementary Information

Supplementary Material 1.

## Data Availability

All data supporting the findings of this study are available in the Open Science Framework (OSF) repository, as indicated and fully accessible through the link provided in the Methods section of this manuscript.
