# Supplementary material for: Systemic and oral adverse effects of semaglutide reported in randomized controlled trials: a scoping review
Source: BMC Oral Health. 2026 Nov 14;26:2264. doi: 10.1186/s12903-026-10305-6 (PMC13769164; doi:10.1186/s12903-026-10305-6)
Supplement: Supplementary file 1 [file 12903_2026_10305_MOESM1_ESM.docx]

Search strategy applied for each database.

| **Database** | **Date** | **Filters** | **Terms** | **Results** |
| --- | --- | --- | --- | --- |
| PubMed | 18.07.2025 | 5 years; RCT | (semaglutide OR “glucagon-like peptide-1 receptor agonist” OR GLP-1RA) AND ("adverse effects" OR "side effects" OR "adverse events") | 300 |
| Web of Science | 18.07.2025 | 2020-2025; articles | (semaglutide OR “glucagon-like peptide-1 receptor agonist” OR GLP-1RA) AND ("adverse effects" OR "side effects" OR "adverse events") | 18 |
| Scopus | 18.07.2025 | 2020-present; RCT | (semaglutide OR “glucagon-like peptide-1 receptor agonist” OR GLP-1RA) AND ("adverse effects" OR "side effects" OR "adverse events") | 312 |
